# Preserved Discrimination Performance and Neural Processing during Crossmodal Attention in Aging

**DOI:** 10.1371/journal.pone.0081894

**Published:** 2013-11-20

**Authors:** Jyoti Mishra, Adam Gazzaley

**Affiliations:** Department of Neurology, Physiology and Psychiatry, University of California, San Francisco, San Francisco, California, United States of America; Hospital General Dr. Manuel Gea González, Mexico

## Abstract

In a recent study in younger adults (19-29 year olds) we showed evidence that distributed audiovisual attention resulted in improved discrimination performance for audiovisual stimuli compared to focused visual attention. Here, we extend our findings to healthy older adults (60-90 year olds), showing that performance benefits of distributed audiovisual attention in this population match those of younger adults. Specifically, improved performance was revealed in faster response times for semantically congruent audiovisual stimuli during distributed relative to focused visual attention, without any differences in accuracy. For semantically incongruent stimuli, discrimination accuracy was significantly improved during distributed relative to focused attention. Furthermore, event-related neural processing showed intact crossmodal integration in higher performing older adults similar to younger adults. Thus, there was insufficient evidence to support an age-related deficit in crossmodal attention.

## Introduction

Integration of concurrently presented information in the auditory and visual modalities is a vital feature of real-world multisensory processing. Such crossmodal integration intimately interacts with attention, which dictates selective focus in the visual and/or auditory modalities depending on relevance to top-down goals. How aging impacts crossmodal interactions with attention has been investigated in very few studies, and is the focus of the present research. 

While unisensory auditory and visual processing, as well as cognitive functions such as attention, are known to degrade with age [2-7], bisensory or crossmodal performance in aging has been evidenced to be equivalent to or even enhanced relative to performance in younger adults [8-13] with few exceptions [14,15]. Amongst these, only two studies have explored the interaction between crossmodal integration and attention on aging behavior, and found evidence for intact crossmodal attention in aging [11,12]. Of note, no study to-date has investigated the neural underpinnings of crossmodal attention in aging. Uniquely, here we investigated the impact of attention on crossmodal performance accuracy and response times (RTs) in aging, simultaneous with the underlying neurophysiology of these interactions as measured by event-related potential (ERP) recordings. 

The present study utilized an experimental paradigm that we recently used to investigate crossmodal attention in younger adults [1]. It incorporated two attentional manipulations: (1) attention focused on one sensory modality: visual, and (2) attention distributed across the visual and auditory modalities. In addition, there were two audiovisual (av) stimulation conditions: (1) semantically congruent (av) words (e.g. concurrent visual ‘dog’ and auditory word ‘dog’) and (2) semantically incongruent (av) words (e.g. concurrent visual word ‘dog’ and auditory word ‘bed’). The semantically incongruent stimuli were presented to generate interference between the concurrent visual and auditory stimuli and to study the impact of this semantic interference on behavior and underlying neural processing. All (av) stimuli were presented interspersed in a stream of visual only (v) and auditory only (a) stimuli. Participants discriminated animal targets from non-animal stimuli. Thus, during focused visual attention, participants ignored all auditory stimuli, and reported animals that occurred in the (v) stream and in the visual component of the (av) stream. During distributed audiovisual attention, participants reported animal targets in the (v), (a) and (av) streams. Note, that in this experiment, there was always a single top-down goal, to detect animal targets, but this goal could either be exclusive to the visual modality (focused visual attention condition) or distributed across audiovisual modalities (distributed audiovisual attention condition). Also note that we preferred to use ‘distributed attention’ in our terminology distinct from ‘divided attention’, as the latter has often been used to refer to attention divided across more than one top-down goal in more than one sensory modality (e.g., discriminating animals in the visual modality and vehicles in the auditory modality) [16]. Due to experimental time constraints a third attention condition, attention exclusively focused on the auditory modality was not assessed.

In Mishra and Gazzaley [1], we showed that younger adults exhibited performance benefits under distributed audiovisual attention relative to focused visual attention for both semantically congruent and incongruent (av) stimulation. Faster RTs with uncompromised discrimination accuracies were exhibited under distributed attention for congruent (av) stimuli and improved discrimination accuracies with uncompromised RTs were observed under distributed attention for incongruent (av) stimuli. Early sensori-neural processing of the auditory and visual constituents of (av) stimulation were calculated in (av-v) and (av-a) ERP difference waves, respectively. For both auditory and visual constituent processing, consistent neural signatures emerged as reduced processing amplitudes under distributed audiovisual relative to focused visual attention. These processing results generalized across congruent and incongruent audiovisual stimulus settings. 

Here, we conducted the above described experiment in older adults. Given that prior behavioral research has shown both intact crossmodal integration and crossmodal attention in aging, we hypothesized age-invariant or even enhanced behavioral performance with aging in the present study. Specifically, this hypothesis predicted improved performance under distributed audiovisual relative to focused visual attention in older adults similar to that found in younger adults. Note that these results were expected based on the theoretical framework that performance is most improved when top-down attention resources are focused on all sensory information relevant to a given task goal, as would occur under the distributed audiovisual attention condition. In contrast during focused visual attention, stimuli in the ignored auditory modality may capture bottom-up attention, generating processing that competes with the top-down attention-related goal processing [17-20]. Indeed neural findings in our previous experiment in young adults confirmed that greater sensory processing with less concomitant behavioral benefit, occurs during focused visual attention relative to distributed audiovisual attention [1]. Thus we expected similar behavioral as well as neural outcomes in older relative to younger adults. While a neural investigation of crossmodal attention in aging has not been done before, neurophysiological studies of crossmodal integration suggest divergent neural processing in younger and older adults in age group averaged data [13,15,21]. If this is indeed the case in the present study, we shall further explore if at least high performing older adults, as differentiated from low performers based on median performance splits, show similar neural processing as younger adults [6].

## Materials and Methods

### Participants

Twenty-two healthy older adults (mean age 68.5 years; range 60-82 years; 9 females) gave written informed consent to participate in the study; study procedures and written consent information were approved by the Committee on Human Research at the University of California in San Francisco (Approval # H53666-28283-05). All participants were screened to ensure they had no history of neurological, psychiatric or vascular disease, were not depressed, and were not taking any psychotropic medications, and had a minimum of 12 years of education. Participants were additionally screened using a 12 multiple-choice questionnaire to document no hearing problems in daily life situations. Prior to the experiment, all participants were examined for normal or corrected-to-normal vision using a Snellen chart, and for normal hearing tested in both ears in the 250 Hz - 6 kHz frequency range as estimated by an audiometry software application UHear©. Individuals with poorer hearing sensitivities than in the ‘mild loss’ range as per UHear© results, were excluded from the study, thus controlling for presbycusis. Data from a cohort of 20 younger participants (mean age 23.4 years, range 19–29 years, 10 females) who previously engaged in the same experiment were utilized for age-group comparisons [1].

### Neuropsychological testing

Prior to the experiment, older adults were administered a battery of thirteen neuropsychological tests. Participants were required to score within two standard deviations of published age-matched normative values on these tests to qualify as healthy older adults for study inclusion; these standard inclusion criteria have been used in all our prior healthy aging studies [5-7,22-27]. The neuropsychological evaluation consisted of tests designed to assess general intellectual function [28], verbal learning (CVLT-II), geriatric depression (GDS), visual-spatial function (modiﬁed Rey-Osterrieth ﬁgure), visual-episodic memory (memory for details of a modiﬁed Rey-Osterrieth Complex Figure (ROCF [29,30]), visual-motor sequencing (trail making tests A and B), phonemic ﬂuency (words beginning with the letter ‘D’), semantic ﬂuency (animals), calculation ability (arithmetic), executive functioning [31], working memory and incidental recall, backward digit span and digit symbol, and WAIS-R. 

### Stimuli & Experimental procedure

Stimuli were presented on Presentation software (Neurobehavioral Systems, Inc.) run on a Dell Optiplex GX620 with a 22” Mitsubishi Diamond Pro 2040U CRT monitor. Participants were seated with a chin rest in a dark room 80 cm from the monitor. Visual stimuli (v), were words presented as black text in Arial font in a grey square sized 4.8° at the fovea. Auditory words (a), were spoken in a male voice, normalized and equated in average power spectral density, and presented to participants at a comfortable sound level of 65dB SPL using insert earphones (Cortech Solutions, LLC). Prior to the experiment participants were presented with all auditory stimuli once, which they repeated to ensure 100% word recognition. All spoken and printed word nouns were simple, mostly monosyllabic everyday usage words e.g. tree, rock, vase, bike, tile, book, plate, soda, ice, boat etc. The experiment used 116 unique written and corresponding spoken words as visual and auditory stimuli, respectively; of these 46 words were animal names (cat, chimp, cow, deer, bear, hippo, dog, rat, toad, fish etc.) and served as targets. Visual stimuli were presented for a duration of 100 ms, all auditory presentations had a 250 ms duration, and audiovisual stimuli (av) had simultaneous onset of the auditory and visual stimulus constituents; all participants perceived the audiovisual stimuli as spatio-temporally aligned. The spoken and written words were identical for congruent (av) stimuli and non-identical for incongruent (av) stimuli. Each experimental run consisted of 360 randomized stimuli (shuffled from the set of 116 unique stimuli), with an equivalent 120 (v) alone, (a) alone and (av) stimulus presentations. The inter-stimulus interval for all stimulus types was jittered at 800-1100 ms. Each experimental block run thus lasted 6 min, with a few seconds of a self-paced break available to participants every quarter block. Stimuli were randomized at each block quarter to ensure equivalent distribution of (a), (v) and (av) stimuli in each quarter. 

**Figure 1:**
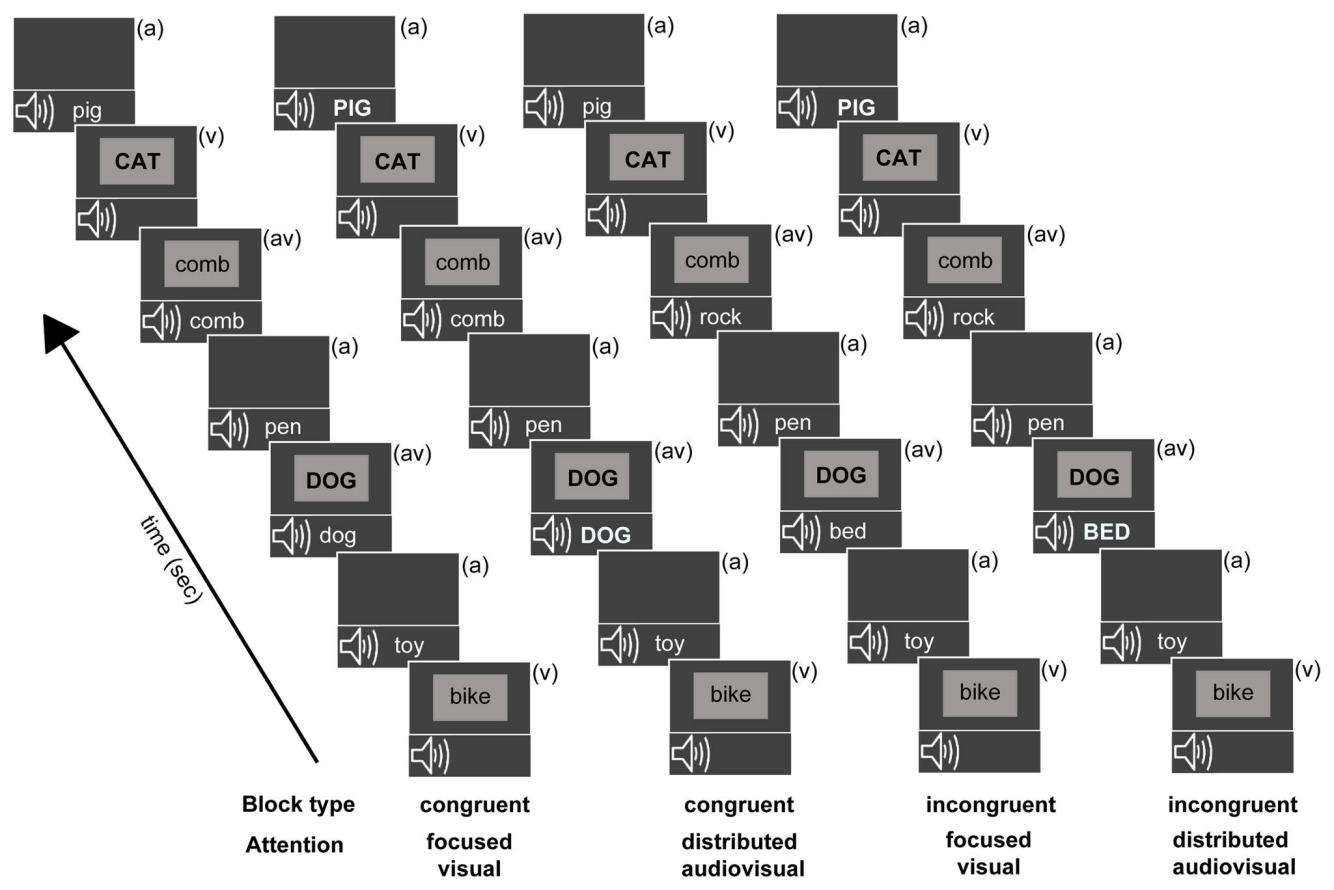
Overview of experimental block design. All blocks consisted of randomly interspersed auditory only (a), visual only (v), and simultaneous audiovisual (av) stimuli, labeled in each frame. The auditory and visual constituent stimuli of audiovisual trials matched during the two congruent blocks, and did not match on incongruent blocks. Target stimuli (animal words) in each block stream are depicted in uppercase (though they did not differ in actual salience during the experiment). During the focused visual attention blocks, participants detected visual animal word targets occurring in either the (v) or (av) stream. During the distributed audiovisual attention blocks, participants detected animal targets occurring in either of three stimulus streams.

There were four unique block types presented in two sets of four blocks each (Figure 1), with block order randomly shuffled in each of the two set repeats: Block type 1: Congruent - Focused Visual; Block type 2: Congruent - Distributed Audiovisual; Block type 3: Incongruent - Focused Visual; Block type 4: Incongruent - Distributed Audiovisual. Participants were briefed as per the upcoming block type, about the attention requirements (focused vs. distributed) as well as stimulus congruency (congruent vs. incongruent), before each block presentation. Block type (1) had congruent (av) stimuli and participants were instructed to focus attention only on the visual stream and respond with a button press to visual animal targets, whether appearing as (v) alone or (av) stimuli (congruent focused visual attention block). In block type (2) (av) stimuli were again congruent and participants were instructed to distribute attention across both auditory and visual modalities and detect all animal names, appearing in the (v), (a) and (av) streams (congruent distributed audiovisual attention block). In block type (3) (av) stimuli were incongruent and participants were instructed to focus attention on the visual stream only and respond to visual animal targets appearing alone or co-occurring with a conflicting non-animal auditory stimulus (incongruent focused visual attention block). Lastly, in block type (4) (av) stimuli were incongruent and participants distributed attention to both (a) and (v) stimuli detecting animal names in the (v), (a) and incongruent (av) stream (incongruent distributed audiovisual attention block). Note that Focused Auditory block types were not included in the experiment in order to constrain the number of experimental manipulations in a single session and provide high quality behavioral and neural data minimally contaminated by fatigue effects.

Targets in the (a), (v), or (av) streams appeared at 20% probability. To further clarify, for the (av) stream in congruent blocks ((1) and (2)), visual animal targets were paired with identical auditory animal targets, while in incongruent blocks ((3) and (4)) visual animal targets were paired with auditory non-animal stimuli i.e. there were no visual non-animal stimuli paired with auditory animal targets in incongruent blocks ((3) and (4)). The non-target incongruent audiovisual stimuli in blocks (3) and (4) paired non-animal visual stimuli with other non-animal auditory stimuli. These particular aspects of the (av) stimuli pairing were unknown to participants (though, in general, participants knew whether an upcoming block was congruent or incongruent) and maintained the same number of visual constituent targets within the (av) streams across all blocks. Note that performance metrics were obtained for targets in the (v) and (av) streams in all blocks, while performance on targets in the (a) stream was only obtained in the distributed audiovisual attention blocks (2) and (4); targets in the (a) stream in the focused visual attention blocks (1) and (3) were not attended to and did not have associated responses. 

Participants were instructed to fixate at the center of the screen at all times, and were provided feedback as per their average percent correct accuracy and RTs at the end of each block. Speed and accuracy were both emphasized in the behavior, and correct responses were scored within a 200–1200 ms period after stimulus onset. 

### Behavioral Data Analyses

Correct responses to targets in each modality were categorized as ‘hits’ while responses to non-target stimuli in each modality were classified as ‘false alarms’. The hit and false alarm rates were used to derive the discrimination index *d*′ separately in each modality. The normsinv function in Excel was used to generate the inverse of the standard normal cumulative distributions for the proportion hit rate and for the proportion false alarm rate in each modality in each individual. *d'* was calculated as *d'* = normsinv(hit rate) - normsinv(false alarm rate) [32]. As previously analyzed in younger adults [1], the impact of focused vs. distributed attention on audiovisual processing was compared using performance indices, as the difference in performance between (av) and (v) stimuli: (av-v) calculated for both attention manipulations and separately for the congruent and incongruent blocks. As one of the two attention manipulations (*focused visual*) did not contain responses to (a) targets, an (av-a) behavioral comparison and further assessments based on the race model [33] were not possible. Statistical analyses for d’ and RT performance metrics utilized repeated-measures analyses of variance (ANOVAs) with a Greenhouse-Geisser correction when appropriate. ANOVA factors included a between-subjects factor of age and three within-subjects factors of congruency (congruent vs. incongruent), attention (focused vs. distributed) and stimulus type ((av) vs. (v)). Younger adults’ data from Mishra and Gazzaley [1] was used for age comparisons. Post-hoc analyses consisted of two-tailed *t*-tests.

### EEG Data acquisition

Data were recorded during 8 blocks (2 per block type) yielding 192 epochs of data for each standard (v)/ (a)/ (av) stimulus (and 48 epochs per target) per block type. Electrophysiological signals were recorded with a BioSemi ActiveTwo 64-channel EEG acquisition system in conjunction with BioSemi ActiView software (Cortech Solutions, LLC). Signals were amplified and digitized at 1024 Hz with a 24-bit resolution. All electrode offsets were maintained between +/-20 mV.

The three-dimensional coordinates of each electrode and of three fiducial landmarks (the left and right pre-auricular points and the nasion) were determined by means of a BrainSight (Rogue Research, Inc.) spatial digitizer. The mean Cartesian coordinates for each site were averaged across all subjects and used for topographic mapping and source localization procedures.

### EEG data analysis

Raw EEG data were digitally re-referenced off-line to the average of the left and right mastoids. Eye artifacts were removed through independent component analyses by excluding components consistent with topographies for blinks and eye movements and the electro-oculogram time-series. Data were high-pass filtered at 0.1 Hz to exclude ultraslow DC drifts. This preprocessing was conducted in the Matlab (The Mathworks, Inc.) EEGLab toolbox (Swartz Center for Computational Neuroscience, UC San Diego). Further data analyses were performed using custom ERPSS software (Event-Related Potential Software System, UC San Diego). ERPs were analyzed for the 80% standard (non-animal) (v), (a) and (av) stimuli. There were too few target trials to analyze target-related ERPs. Signals were averaged in 500 ms epochs with a 100 ms pre-stimulus interval. The averages were digitally low-pass filtered with a Gaussian finite impulse function (3 dB attenuation at 46 Hz) to remove high-frequency noise produced by muscle movements and external electrical sources. Epochs that exceeded a voltage threshold of +/-75 µV were rejected. 

Components of interest were quantified in the 0-300 ms ERPs over distinct electrode sets that corresponded to sites at which component peak amplitudes were maximal. Visual constituent processing of (av) stimulation was quantified in (av-a) difference waves over occipital sites corresponding to the peak topographies of the P1 and N1 latency components (P1: PO3/4, PO7/8, O_1/2_, N1: PO7/8, P7/P8). Auditory constituent processing was quantified in (av-v) difference waves over fronto-central electrodes corresponding to peak topographies of the auditory P2 component (F_1/2_, FC_1/2_, Fz, FCz). Statistical analyses for ERP components utilized repeated-measures analyses of variance (ANOVAs) with a Greenhouse-Geisser correction when appropriate. Post-hoc analyses consisted of two-tailed *t*-tests. This ERP component analysis was additionally confirmed by conducting running point-wise two-tailed paired t-tests at all scalp electrode sites. In this analysis, a significant difference is considered if at least 10 consecutive data points meet the 0.05 alpha criterion and is a suitable alternative to Bonferroni correction for multiple comparisons [34-36]. This analysis did not yield any new effects other than the components of interest described above. 

Of note, here we refrained from analyses of later processes (> 300 ms post-stimulus onset) as it is not easy to distinguish whether such processes reflect a sensory/ multisensory contribution or decision making/ response selection processes that are active at these latencies. 

### Modeling of ERP sources 

Inverse source modeling was performed to estimate the intracranial generators of the components within the grand-averaged difference waves that represented significant modulations in congruent and incongruent multisensory processing. Source locations were estimated by distributed linear inverse solutions based on a local auto-regressive average (LAURA [37]). LAURA estimates three-dimensional current density distributions using a realistic head model with a solution space of 4024 nodes equally distributed within the gray matter of the average template brain of the Montreal Neurological Institute. It makes no a priori assumptions regarding the number of sources or their locations and can deal with multiple simultaneously active sources [38]. LAURA analyses were implemented using CARTOOL software by Denis Brunet (http://sites.google.com/site/fbmlab/cartool). To ascertain the anatomical brain regions giving rise to the difference wave components, the current source distributions estimated by LAURA were transformed into the standardized Montreal Neurological Institute (MNI) coordinate system using SPM5 software (Wellcome Department of Imaging Neuroscience, London, England).

## Results

**Figure 2:**
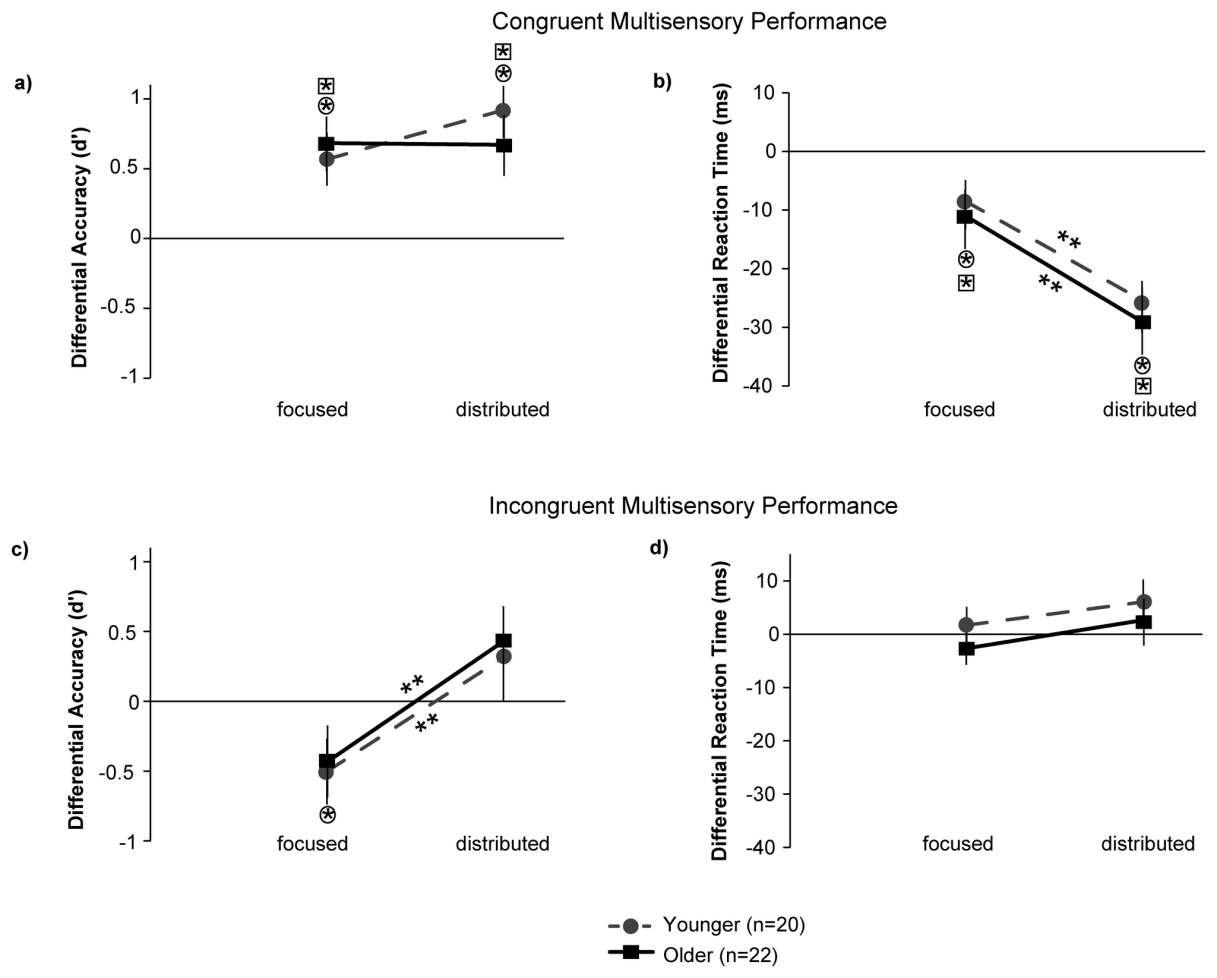
Behavioral performance during the focused and distributed attention conditions for (av) target stimuli normalized relative to performance on (v) targets. Measures are shown as differential d’ (**a**,**c**) and differential RTs (**b**,**d**) to depict (av-v) performance. Asterisks on plotted points (square and circle enclosed asterisks for older and younger adult data, respectively) represent significant (av) vs. (v) performance differences for that attention condition. Asterisks on trend lines indicate significant performance differences between the focused and distributed attention conditions.

Discrimination index (d’) and response times (RT (ms)) for (v) and (av) target stimuli during *Focused Visual Attention* blocks, and for (v), (a) and (av) target stimuli during *Distributed Audiovisual Attention* blocks are shown in Table 1. Younger adults’ data are from Mishra and Gazzaley [1]. These data were used to calculate (av-v) performance indices for each individual from each block type, as previously done for younger adults. (av-v) indices especially for RTs provided an adjustment for overall speed of processing differences across individuals and age groups. Figure 2 shows (av-v) d’ and RT metrics for distributed attention relative to focused attention trials (the line connecting data points in each graph portrays the relative performance between the two attention manipulations); performance indices in the younger adult cohort are shown for comparison with dashed trend lines. 

**Table 1:** Details of behavioral measures observed for target stimuli during the four blocked tasks, 1: congruent stimuli & focused visual attention, 2: congruent stimuli & distributed audiovisual attention, 3: incongruent stimuli & focused visual attention, and 4: incongruent stimuli & distributed audiovisual attention. Values are means +/- standard errors of mean. (v) = visual, (av) = audiovisual, and (a) = auditory. Younger adults’ data were from Mishra and Gazzaley [1].

BlockType	Target	d’ (sem) Older	d’ (sem) Younger	Hits % (sem) Older	Hits % (sem) Younger	False alarm % (sem) Older	False alarm % (sem) Younger	RT (ms) (sem) Older	RT (ms) (sem) Younger
1	(v)	4.8 (0.2)	5.2 (0.2)	96.9 (0.9)	97.5 (0.7)	0.5 (0.1)	0.5 (0.1)	599 (11)	554 (9)
	(av)	5.4 (0.2)	5.7 (0.2)	97.8 (0.9)	98.3 (0.8)	0.5 (0.1)	0.5 (0.1)	587 (10)	545 (9)
2	(v)	4.6 (0.2)	5.0 (0.2)	97.9 (0.5)	97.5 (0.6)	0.8 (0.1)	0.8 (0.1)	598 (13)	548 (7)
	(av)	5.1 (0.2)	5.9 (0.2)	98.3 (0.6)	99.5 (0.3)	0.9 (0.2)	0.9 (0.2)	568 (11)	523 (8)
	(a)	4.0 (0.3)	4.1 (0.2)	87.9 (2.4)	90.1 (1.8)	0.5 (0.1)	0.5 (0.1)	749 (17)	680 (12)
3	(v)	5.5 (0.2)	5.4 (0.2)	98.4 (0.7)	97.3 (1.2)	0.6 (0.1)	0.6 (0.1)	585 (11)	548 (9)
	(av)	5.0 (0.2)	4.9 (0.2)	97.9 (0.7)	96.9 (0.7)	0.7 (0.1)	0.7 (0.1)	582 (10)	550 (8)
4	(v)	4.9 (0.3)	5.1 (0.2)	96.9 (0.8)	97.6 (0.7)	1.0 (0.3)	1.0 (0.3)	576 (10)	538 (8)
	(av)	5.5 (0.2)	5.4 (0.2)	98.5 (0.7)	98.5 (0.5)	0.9 (0.2)	0.9 (0.2)	578 (10)	544 (9)
	(a)	3.8 (0.3)	4.4 (0.2)	84.9 (3.9)	91.7 (2.0)	0.5 (0.1)	0.5 (0.1)	740 (18)	681 (11)

2x2x2x2 repeated measures ANOVAs with between-subjects factor of age and within-subjects factors of congruency (congruent vs. incongruent), attention (focused vs. distributed) and stimulus type ((av) vs. (v)) were used to assess age-related differences in performance. Importantly, significant interactions of age x attention x stimulus type or age x congruency x attention x stimulus type would reveal differential influences of crossmodal attention with aging. Other age interactions devoid of the stimulus type factor would indicate age differences in semantic processing (age x congruency) or differences in attention (age x attention) or differences in attending under certain semantic congruency (age x congruency x attention), but crucially no differences in crossmodal attention. 

The ANOVA on the accuracy measures (d’) showed no main effect of age (p=0.35), no main effect of congruency (p=0.14) and no main effect of attention (p=0.57). A main effect of stimulus type showed greater accuracy for (av) than (v) targets (F(1,40)=13.57, p=0.0007). Importantly, there were no interactions between age and other factors that would suggest changes in crossmodal attention with aging (age x attention x stimulus type: p=0.64, age x congruency x attention x stimulus type: p=0.55). The only age interaction found to be significant was age x congruency x attention (F(1,40)=4.85, p=0.03), which showed that collapsed across all stimulus streams, older adults had noticeably worse performance than younger adults, specifically during distributed attention in the incongruent block; parsed further in the next paragraph. More results for the 2x2x2x2 d' ANOVA were a significant attention x stimulus type interaction, which showed that (av) vs. (v) accuracies were better under distributed than focused attention (F(1,40)=9.15, p=0.004). A significant congruency x attention x stimulus type interaction also emerged (F(1,40)=4.05, p=0.05); post-hoc t-tests showed that this interaction was driven by the d' results for incongruent targets for which the (av-v) d' index improved from a negative to a positive index for focused vs. distributed attention (p=0.003, Figure 2c), while the (av-v) d' index was not modulated with attention for congruent targets (p=0.39, Figure 2a). Note that a negative (av-v) d’ index indicates poor performance accuracy on incongruent audiovisual relative to visual only trials, which occurred due to crossmodal interference during focused visual attention. This interference was remarkably recovered during distributed attention as observed in the positive (av-v) d' indices. Also note that although Figure 2a suggests an age interaction on congruent d', this was also explicitly confirmed to be non-significant (p=0.37). 

The significant age x congruency x attention interaction for d' was further evaluated separately for target hits and non-targets false alarms. For both hits and false alarms 2x2x2x2 ANOVAs were conducted with between-subjects factors of age and within subjects factors of congruency, attention and stimulus type. No main age effects or age interactions emerged for hits. False alarms, however, showed a main effect of age (F(1,40)=10.73, p=0.002) with greater false alarms in older adults, an age x congruency interaction (F(1,40)=5.46, p=0.02) with greater false alarms in older adults in the incongruent block, an age x attention interaction (F(1,40)= 10.45, p=0.002) with greater false alarms in older adults during distributed attention, and finally an age x congruency x attention interaction (F1,40)=4.59, p=0.04) with most false alarms in older adults under distributed attention in the incongruent block. Notably, false alarms did not show any significant age interactions with stimulus type or with combinations of stimulus type and other factors i.e, false alarms for audiovisual relative to visual targets were not differentially impacted with aging, overall or in any stimulus congruency or attention setting.

A 2x2x2x2 ANOVA on RTs showed a main effect of age (F(1,40)=9.837, p=0.003) with significantly slower RTs in older adults, no main effect of congruency (p=0.38), a main effect of attention with faster RTs during distributed attention (F(1,40)=13.34, p=0.0007) and a main effect of stimulus type with faster RTs to audiovisual stimuli (F(1,40)=16.87, p=0.0002). Consistent with d' results there were no interactions between age and any other factors to suggest differential crossmodal attention with aging (age x attention x stimulus type: p=0.99, age x congruency x attention x stimulus type: p=0.73), and age showed no significant interactions with any other combination of within subjects' factors. A significant attention x stimulus type interaction showed that (av) vs. (v) RTs were faster during distributed than focused attention (F(1,40)=6.32, p=0.02). A significant congruency x attention x stimulus type interaction also emerged (F(1,40)=43.59, p<0.0001); post-hoc t-tests showed this interaction was driven by the more negative (av-v) RT index (i.e., faster RTs) during distributed relative to focused attention for congruent targets (p<0.0001, Figure 2b), while incongruent targets did not show this modulation (p=0.1, Figure 2d). 

Thus, overall, irrespective of age, individuals showed a crossmodal RT advantage for congruent stimuli and a crossmodal d' advantage for incongruent stimuli during distributed audiovisual relative to focused visual attention. The effect size of the congruent crossmodal RT gain with distributed attention in older adults was comparable to the effect size in younger adults (0.71 vs. 0.66). Similarly, the effect sizes of the incongruent crossmodal d' gain with distributed attention in both age groups were large (older: 0.74, younger: 1.09). Notably, false alarms showed age differences, but not differentially for crossmodal (av) vs. unimodal (v) stimuli, hence, differential (av-v) d' performance indices were preserved with aging (Figure 2a, c), highlighting a crossmodal performance advantage in the face of general age-related inhibitory deficits that presented as significantly more false alarms. 

Finally, we analyzed the performance data on the unisensory targets. (v) targets that served as a baseline measure (horizontal zero line: Figure 2) were subjected to 2x2x2 ANOVAs for d’ and RT measures respectively, with age as the between-subjects factor, and block congruency (congruent vs. incongruent) and attention (focused vs. distributed) as within-subjects factors. The ANOVA on d’ measures showed neither a main effect of age (p=0.37) nor any interaction of age with congruency (p=0.75) or age with attention (p=0.71). The ANOVA on (v) target RTs showed a main effect of age (F(1,40)=10.11, p=0.003), but again no interactions between age and congruency (p=0.12) or age and attention (p=0.71) were found. Performance on the isolated auditory (a) targets, which only occurred in the distributed attention conditions, was compared in 2x2 ANOVAs with age as a between-subject factor and block congruency (congruent vs. incongruent) as a within-subject factor. Auditory d’ accuracies showed no main effect of age (p=0.24) nor any age x congruency interaction (p=0.58). For auditory RTs, the 2x2 ANOVA yielded a main effect of age (F(1,40)=9.96,p=0.003) with slower RTs in older adults, and no age x congruency interaction (p=0.39). Overall, these analyses showed that unisensory targets did not show differential age interactions with attention and semantic congruency.

Of note, collapsed across all stimulus conditions in the experiment, d' measures had a larger coefficient of variation (CV) in older (0.17) than younger (0.12) adults. RT measures, also collapsed across all stimuli, showed greater CV in older (0.085) than younger (0.046) adults. These results align with the well-documented finding of greater behavioral instability with aging [39-42]. 

### Event-related Potential (ERP) Responses

#### Effects of attention on congruent audiovisual processing

**Figure 3:**
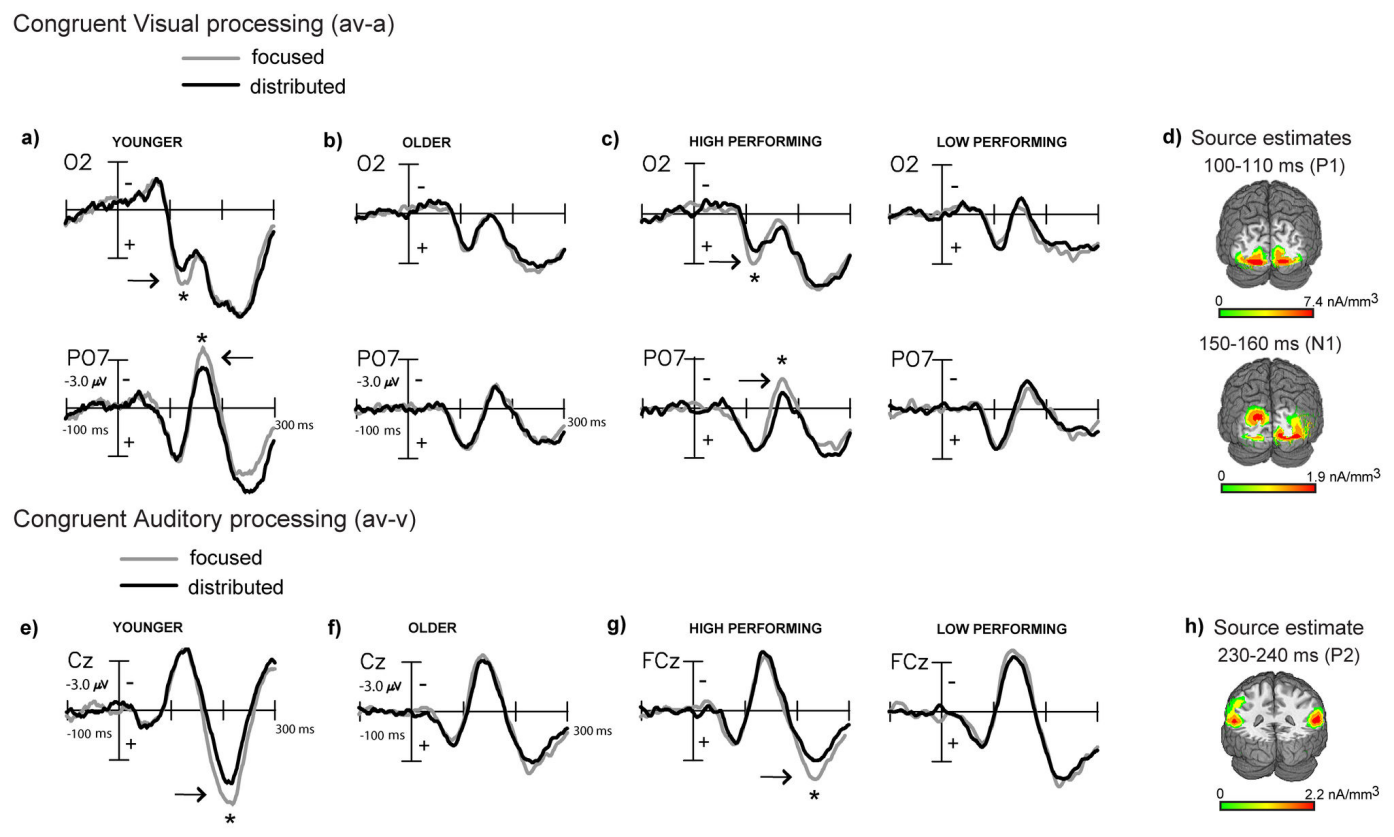
Grand-averaged difference waves (n=22) depicting multisensory processing during the congruent trials compared for the focused and distributed attention conditions. **a**) Extracted processing for the visual constituent of multisensory stimulation (av-a) at occipital sites (O2 and PO7) showing significant visual P1 and N1 latency amplitude differences in younger adults, (**b**) no differences in older adults, (**c**) differences similar to younger adults in high, but not low performing older adults, and (**d**) source estimates of the P1 and N1 latency modulations. (**e**-**h**) Parallel effects obtained for processing of the auditory constituent of multisensory stimulation (av-v) showing attention related differences at P2 latencies.

Behaviorally, we found that distributed audiovisual attention improved discrimination performance relative to focused visual attention for congruent audiovisual stimuli via more rapid RTs (Figure 2b). This was consistently found in both younger and older adults. Previously we had investigated the underlying neural measures in younger adults by calculating the event-related processing of the visual and auditory constituents of the congruent (av) stimuli under distributed and focused attention. Visual constituent processing was obtained at occipital sites by subtracting the auditory alone ERP from the audiovisual ERP within each attention block [43,44]. In younger adults, this (av-a) difference wave revealed significantly reduced signal amplitudes at latencies of 130-140 ms and 160-190 ms in the distributed relative to focused attention condition (Figure 3a (positive µV plotted below horizontal axis)). Source estimates of the extracted visual processing signal at 130-140 ms and at 160-190 ms showed neural generators in extrastriate visual cortex in the region of BA 19, which respectively resembled the P1 and N1 components commonly elicited in the visual evoked potential [45-47]. 

A similar comparison as above in older adults showed exactly overlapping (av-a) difference waves under focused visual and distributed audiovisual attention (Figure 3b). To further unravel the neural data, we assessed the (av-a) difference waves for the higher and lower performing subgroups of older adults split by median RT gain from focused to distributed attention (Figure 3c, [6]). This performance split revealed an early P1-like effect at 100-110 ms with reduced amplitudes under distributed relative to focused attention observed in high performing older adults, similar to the early 130-140 ms latency results obtained in younger adults (age (younger vs. high performing older) x attention (focused vs. distributed): F(1,29)=0.02, p>0.8). Low performing adults did not show this signal modulation (age (younger vs. low performing older) x attention: F(1,29)=4.19, p=0.05)). Similar to younger adults, high performing older adults also showed an N1-like modulation at 150-160 ms (age (younger vs. high performing older) x attention (focused vs. distributed): F(1,29)=1.05, p=0.3), while low performing older adults did not exhibit this effect (age (younger vs. low performing older) x attention: F(1,29)=12.29, p=0.002). These P1 and N1-like latency modulations in older adults localized to extrastriate visual cortex, BA18/19 (Figure 3d) in close proximity to their counterpart component sources found in the younger adult difference waves (MNI co-ordinates of the peaks of the source clusters in table 2). 

**Table 2:** MNI coordinates of the peaks of the source clusters as estimated in LAURA at relevant component latencies identified in the extracted visual (av-a) and extracted auditory (av-v) difference waveforms for congruent and incongruent blocks. All sources were modeled for difference waves in the focused visual attention condition in high performing older adults. Younger adults’ data were from Mishra and Gazzaley [1].

Block type	Difference wave	Older latency		Older		Younger latency		Younger	
		(ms)	x (mm)	y (mm)	z (mm)	(ms)	x (mm)	y (mm)	z (mm)
Congruent	(av-a)	100-110	±24	-77	-2	130-140	±29	-71	-1
	(av-a)	150-160	±26	-83	-7	160-190	±27	-75	-4
	(av-v)	230-240	±53	-37	15	175-225	±56	-33	+7
Incongruent	(av-a)	110-120	±12	-78	4	110-120	±30	-71	-2
	(av-v)	235-245	±54	-33	7	110-120	±58	-35	+4

Previously in younger adults, we compared auditory constituent processing for the congruent (av) stimuli in (av-v) difference waves calculated during distributed audiovisual vs. focused visual attention. This analysis in younger adults showed a significant positive component difference at 175-225 ms or P200, which was larger when the auditory information was task-irrelevant during focused visual attention relative to distributed audiovisual attention (Figure 3e, [1]). Moreover, this (av-v) processing difference directly correlated with the (av-v) RT improvement observed for distributed vs. focused attention. 

Grand-averaged (av-v) difference waves in older adults did not show any processing differences across distributed vs. focused attention (Figure 3f). Again, the RT based performance split in older adults revealed a P2 positivity peaking at 230-240 ms latency that was larger in focused relative to distributed attention in high performing adults akin to the P200 findings in younger adults (age (younger vs. high performing older) x attention: F(1,29)=0.01, p>0.9), while low performing older adults did not show this P2 latency processing difference (age (younger vs. low performing older) x attention: F(1,29) = 4.58, p= 0.04) (Figure 3g). This 230-240 ms P2 positivity localized to superior temporal gyrus (STG, Figure 3h) in close proximity to the P200 source in younger adults (MNI co-ordinates of the peak of the source cluster in table 2). 

Overall, these results consistently show that at least for high performing older adults, the neural modulations were similar to those observed in younger adults. Of note, the median RT performance splits revealed that the congruent (av-v) RT facilitation during distributed vs. focused attention was limited to the high performing older participants (high performing: t(10)=7.91, p<0.0001, low performing: t(10)=0.67, p=0.52). No neurobehavioral correlations emerged between the ERP component modulations in congruent visual/auditory processing in older adults and the congruent crossmodal RT facilitation with distributed attention.

#### Effects of attention on incongruent audiovisual processing

**Figure 4:**
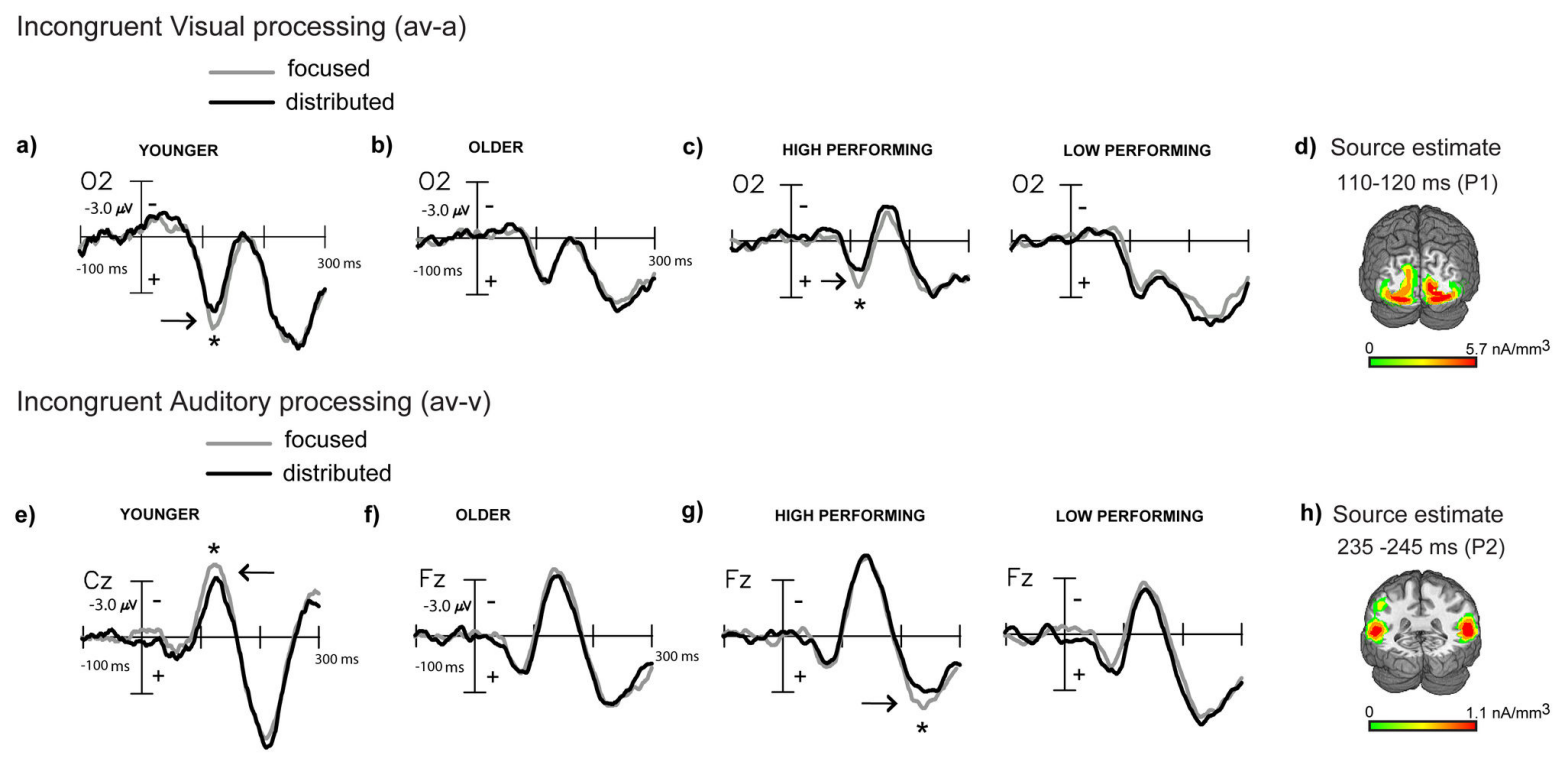
Grand-averaged difference waves (n=22) depicting multisensory processing during the incongruent trials compared for the focused and distributed attention conditions. **a**) Extracted processing for the visual constituent of multisensory stimulation (av-a) at occipital site (O2) showing significant visual P1 latency amplitude differences in younger adults, (**b**) no differences in older adults, (**c**) differences similar to younger adults in high, but not low performing older adults, and (**d**) source estimates of the P1 latency modulation. (**e**-**h**) Parallel effects obtained for processing of the auditory constituent of multisensory stimulation (av-v) showing attention related differences at P2 latencies.

In both younger and older adults we found that distributed attention improved (av-v) accuracies for incongruent audiovisual stimuli relative to focused visual attention (Figure 2c). Parallel to the ERP analysis for congruent stimuli, we first analyzed the visual constituent of incongruent (av) stimulus processing in (av-a) difference waves obtained for both focused and distributed attention conditions. In younger adults, the incongruent (av-a) difference waves had significantly reduced signal amplitudes at 110-130 ms during distributed relative to focused attention (Figure 4a). This P1-like component localized to extrastriate visual cortex (BA 19), in proximity to the P1 latency source in the congruent (av-a) difference waves. Again in older adults, the grand-averaged (av-a) difference waves yielded no difference across the two attention manipulations (Figure 4b). In this case, median performance splits based on d’ accuracy improvements across distributed relative to focused attention revealed a 110-120 ms processing difference with reduced amplitudes during distributed attention observed in high performing older adults akin to younger adults (age (younger vs. high performing older) x attention: F(1,29)=0.13, p=0.72), but not in low performing older adults (age (younger vs. low performing older) x attention: F(1,29)=4.2, p=0.05) (Figure 4c). This P1 latency difference wave component also localized to extrastriate visual cortex (BA18, Figure 4d) in proximity to the P1 latency source estimates in younger adults (MNI co-ordinates of the peak of the source cluster in table 2).

In younger adults, auditory constituent processing calculated in (av-v) difference waves for incongruent audiovisual stimuli showed significantly reduced amplitudes during distributed relative to focused attention at early 110-120 ms latencies at fronto-central sites that localized to middle temporal gyrus (Figure 4e, [1]). Again, the grand-averaged (av-v) waveforms in older adults were overlapping in the two attention conditions (Figure 4f). d’ accuracy dependent median performance splits revealed a 235-245 ms P2 latency processing difference for high performing, but not low performing older adults as analyzed in ANOVAs with attention as a factor (high performing: F(1,10)=4.9, p=0.05, low performing: F(1,10)=0.97, p=0.3) (Figure 4g). A comparison with younger adults was not possible in this case as the earlier N1 latency component was modulated in the younger age group; although mechanistically for both younger and high performing older adults distributed attention was associated with reduced signal amplitudes relative to focused attention. The P2 latency positivity in older adults localized to superior temporal gyrus (STG, BA22: Figure 4h) in close proximity to the P2 latency source found during congruent multisensory processing above (MNI co-ordinates of the peak of the source cluster in table 2).

Thus, similar to findings in congruent blocks, incongruent audiovisual processing in high performing older adults exhibited reduced processing of the visual and auditory constituents under distributed attention. The median d’ performance split showed that the d’ facilitation during distributed vs. focused attention was highly significant for the high performing older adults (t(10)=6.78, p<0.0001) but only trended towards significance for the low performers (t(10)=2.05, p=0.07). No neurobehavioral correlations emerged between the ERP component modulations in incongruent visual/auditory processing in older adults and the incongruent crossmodal d' facilitation with distributed attention.

Finally, across all neural modulations described above (absolute magnitudes averaged together), the CV in older adults (0.74) was greater than twice that in younger adults (0.35), as also observed for the behavioral d' and RT measures.

## Discussion

In the present study we show parallel crossmodal performance in older and younger adults under distributed audiovisual relative to focused visual attention. Distributed attention generated faster (av) response times without a compromise on accuracy in congruent stimulus settings. Additionally, improved response accuracies with unaffected RTs were observed for incongruent stimuli. Note that incongruent audiovisual stimuli generated semantic interference, which was observed in reduced performance accuracy (d’) for incongruent (av) stimuli relative to unisensory visual stimuli during focused visual attention. These audiovisual relative to visual performance decrements observed under focused attention were resolved during distributed attention. Equivalent crossmodal performance in younger and older adults were revealed by null interactions between the factors of age, attention and stimulus type for both accuracy and RT metrics. Of note, older adults did show significantly greater false alarms and significantly slower RTs overall relative to younger adults, but this did not interact with performance improvements on crossmodal stimuli. Event-related potential recordings during the task further revealed that early sensory processing of the auditory and visual constituents of (av) stimulation were consistently reduced during distributed relative to focused attention. These neurophysiological findings that resembled results in younger adults, were restricted to higher performing older adults, as determined by a median performance split of the gain from focused to distributed attention. Thus, the novel result recently found for younger adults of improved behavioral performance being associated with reduced auditory and visual processing during distributed (av) attention, was replicated here for high performing older adults.

Only two prior aging studies investigated the interaction between attention and crossmodal performance [11,12]. Hugenschmidt et al. [12] studied behavioral differences during unisensory (auditory/ visual) cued vs. uncued attention and showed that older adults, similar to younger adults, exhibit performance benefits when attention is cued. These results were consistent whether the task goals were to discriminate the spatial location or categorical identity of target stimuli. Similar to the present study, Hugenschmidt et al. [11] investigated crossmodal attention by manipulating attention focus either to a single modality (auditory/ visual) or to both sensory modalities. Audiovisual stimuli in this study were always congruent visual color images and color sounds. Similar to results presented here, the authors showed the greatest crossmodal RT advantage during distributed (or divided) attention relative to selective visual/auditory attention, observed in both younger and older adults. Here, we further extend and generalize the findings in [11] by incorporating both congruent and incongruent audiovisual stimuli in our experiment; we show that distributed crossmodal attention significantly improves performance, even for incongruent (i.e., crossmodally interfering) stimuli, and equivalently in younger and older adults. Note that for incongruent (av) stimuli we found evidence for interference in reduced audiovisual relative to visual accuracies only during the focused attention condition. This interference effect was recovered during distributed attention. Overall, our findings are consistent with those of [11], even though we use different stimuli types (congruent and incongruent spoken (a) and written (v) stimuli vs. exclusively congruent spoken (a) and pictorial (v) stimuli in the prior study), different task designs (blocked attention vs. trial by trial cued attention in the prior study) and different response schemes (go-no/go vs. two-alternative forced choice in the prior study). Similar results despite these experimental task differences speak to the robustness and generalizability of these findings. Of note, some prior research has evidenced that crossmodal integration in older adults is not just equivalent to, but may even be enhanced relative to younger adults [9,10,48]. In terms of crossmodal (audiovisual) vs. unimodal (visual) comparisons, here we only find evidence for age-equivalence, but not crossmodal enhancement with aging. However, taking into account the generally higher false alarm rates and slower RTs in older adults, which suggest overall deficits in inhibitory control and processing speed [49,50], it is remarkable that older adults are capable of equivalent crossmodal performance gains comparable to younger adults as observed here. 

To investigate the neural basis of crossmodal attention in older adults, we compared early sensory processing of both the visual and auditory constituents of (av) stimulation using difference wave calculations [1]. Higher performing older adults, as separated from lower performers by median behavior splits, showed neural correlates similar to those previously observed in younger adults. The fact that group-averaged neural data in older participants did not show ERP component modulations could be due to the generally greater variability in the older relative to younger adults' data [39-42]. In high performing older adults, similar to younger adults, the visual constituent of (av) stimulation showed reduced signal amplitudes during distributed relative to focused attention at visual P1 and N1 latencies for congruent stimuli, and only at P1 latencies for incongruent stimuli. That distributed audiovisual attention was associated with reduced visual constituent processing compared to focused visual attention, is consistent with observations of sensory processing under unimodal divided attention [51-55] and with the attentional load theory, which posits that limited attentional resources as available under divided attention, are associated with reduced neural responses [56]. 

The neural signal corresponding to the auditory constituent of (av) stimulation was also found to be reduced during distributed audiovisual relative to focused visual attention for higher performing older adults. For both congruent and incongruent (av) stimuli, this amplitude modulation occurred at P2 component latencies and localized to the superior temporal region – a known site for crossmodal integration [43,57-59]. Results for congruent stimuli matched those in younger adults, however, for incongruent stimuli an earlier N1 latency modulation was observed in younger adults. Of note, the direction of modulation, whether at N1 latencies in younger adults or P2 latencies in older adults, remained the same i.e. consistently reduced signal amplitudes during distributed attention. Given that conventionally in unimodal research, task-relevant attended information is enhanced in neural processing relative to task-irrelevant information, the reduced auditory constituent signal amplitudes during distributed audiovisual attention when auditory information was task-relevant vs. focused visual attention when auditory information was task-irrelevant may be unexpected. However, prior crossmodal studies have shown that during a focused visual attention task, a concurrent stimulus in the auditory modality captures bottom-up attention such that auditory neural processing is enhanced relative to an inattentive baseline [17-20]. We interpret our findings as revealing that during distributed audiovisual attention, top-down control reduces the bottom-up capture by the interfering auditory stream and/or may even suppress the interfering stream, resulting in reduced early auditory processing and better performance accuracies as observed here and in our previous study [1]. We propose that it was *distributed* crossmodal attention that conferred the observed behavioral advantages given that in our experiment the same top-down goal (discrimination of animal targets) was shared across both visual and auditory modalities. Suppression of interfering stimuli and concomitantly reduced sensori-neural processing of such stimuli was possible in this case. However, if top-down attention were truly divided between modalities i.e., monitoring of two distinct top-down goals in the auditory vs. visual modality, hence taxing attentional reserves, no behavioral advantages may be evident as is also supported by studies in younger adults [60,61]. Thus, the results observed here were an outcome of distributed, and not divided crossmodal attention.

A limitation of the present study is the absence of the focused auditory attention condition (i.e, attend auditory and ignore visual stimuli), which could not be included in a single visit experiment due to practical constraints. Although our behavioral data suggested worse performance in the auditory modality (as measured under distributed audiovisual attention) relative to the visual/audiovisual modalities, this was not due to poor audibility as we ensured that all participants had 100% recognition of auditory stimuli prior to initiation of the experiment. In general, this data supported sensory dominance of the visual modality over the auditory modality as previously known [62,63]. Observed visual dominance in humans and its real world relevance was in fact one of our main reasons to choose focused visual attention, instead of focused auditory attention, as the comparison condition relative to distributed attention in our experiment. We predict, however, that a focused auditory attention condition incorporated in a future experiment would elicit worse crossmodal performance relative to distributed audiovisual attention and perhaps also relative to focused visual attention, given that prior studies have evidenced greater negative impacts of visual distractions during auditory attention than auditory distractions during visual attention [64,65]. 

Another unique aspect, but also limitation of the present study was the use of semantic stimuli. It remains possible that non-semantic audiovisual associations, such as non-speech sounds and visual images, do not interact with attention as found here and do not show age-invariant results; this remains to be investigated in future studies. In fact, in younger adults crossmodal attention research has shown that for arbitrary audiovisual stimulus combinations (shapes and tones), there is no behavioral advantage observed when attention is distributed across both modalities [60,61]. Crucially, however, in these studies the stimuli had no inherent associations, and so distributing attention across modalities was akin to more difficult dual-tasking with divided attention between two unique task goals in the auditory and visual domain. Hence, to study the advantages of distributed audiovisual attention for non-speech stimuli, it is recommended that future studies use stimuli that have inherent associations such as animal/ object sounds and images (e.g. 18). Finally, it would be informative to perform magnetic resonance imaging (MRI) and diffusion tensor imaging (DTI) in such studies, especially to reveal differences in neural network connectivity and in structural measures, such as gray matter volume and white matter integrity, between younger vs. older adults and between high vs. low performing older adults [26,27,66-70]. 

Overall, our study corroborates prior age-related behavioral findings of preserved crossmodal attention in aging. Here, we have generalized these results to semantically congruent as well as incongruent audiovisual stimuli. We also found that distributed audiovisual attention, which resulted in improved behavioral performance relative to focused visual attention, was associated with reduced early sensori-neural signals in both vision and audition that localized to visual extrastriate and polysensory temporal cortices, respectively. Overall the neural modulations suggested that early audiovisual event-related sensory processing and its interaction with attention is preserved in aging, at least in high performing older adults. 
